# Reasons for and barriers to biosafety and biosecurity training in health-related organizations in Africa, Middle East and Central Asia: findings from GIBACHT training needs assessments 2018-2019

**DOI:** 10.11604/pamj.2020.37.64.23390

**Published:** 2020-09-16

**Authors:** Elizeus Rutebemberwa, Fortress Yayra Aku, Eva Inam Kayed Al Zein, Hedia Bellali

**Affiliations:** 1 Programs, African Field Epidemiology Training Network, Kampala, Uganda,; 2 Department of Health Policy, Planning and Management, School of Public Health, Makerere University, Kampala, Uganda,; 3 Department of Epidemiology and Biostatistics, School of Public Health, University of Health and Allied Sciences, Hohoe, Ghana,; 4 Jordan Food and Drug Administration, Amman, Jordan,; 5 Abderrahmen Mami Hospital, Ariana, Tunisia,; 6 Medical Faculty of Tunis, Tunis El Manar University, Tunis, Tunisia

**Keywords:** Biosafety, biosecurity, GIBACHT, barriers, Africa, Middle East, Asia

## Abstract

**Introduction:**

the Global-Partnership-Initiated-Biosecurity-Academia for Controlling Health Threats (GIBACHT) consortium conducts a biosafety and biosecurity training for fellows from Africa, the Middle East and Asia. To achieve a multiplier effect, fellows conduct trainings in their own organizations. It was during such trainings that training needs assessments were done assessing reasons for and barriers to biosafety and biosecurity training.

**Methods:**

this was a cross sectional assessment. Trainings were conducted from April to July 2018 and April to June 2019. In 2018, training needs were explored using a structured tool. Responses were coded using manifest content analysis and key issues identified. In 2019, respondents quantified the identified key issues using a Likert scale. Proportions of those who strongly agreed, agreed, neither agreed nor disagreed, disagreed or strongly disagreed were calculated and results presented in tables and charts.

**Results:**

in 2018 and 2019, there were 183 and 191 respondents respectively. About 96% of respondents in 2018 supported training in biosafety and biosecurity citing individual, community and global benefits. Barriers highlighted included governance, financial, human resource, information and infrastructure challenges. In 2019, majority of respondents indicated inadequate guidelines dissemination, lack of financial resources, inadequate personnel, lack of equipped laboratories and lack of instructional materials among major barriers.

**Conclusion:**

support for biosafety and biosecurity training was high though systemic barriers exist. Improving human resource capacity and provision of instructional materials can be achieved through training programs. However, systemic assessments need to be done before each training as different organizations have different barriers.

## Introduction

The COVID-19 outbreak first reported in Wuhan, China in December 2019 had within three months rapidly spread across all continents and was reported in over 200 countries and territories [1]. This highlights the global need for biosafety and biosecurity training which has been underscored over time. An increase in wildlife and human interaction has led to a rise in zoonotic diseases among the emerging infectious diseases [2]. This has been fueled by increasing economic development, urbanization and global travel [3]. There has also been a rise in the number of laboratories engaged in infectious diseases [4]. However, some studies have shown limited awareness of biosecurity or biosafety in laboratory workers in low income countries [5-7].

The Global-Partnership-Initiated-Biosecurity-Academia for Controlling Health Threats (GIBACHT) consortium was formed by the Bernhard Nocht Institute of Tropical Medicine (BNITM), Robert Koch Institute (RKI), Swiss Tropical and Public Health Institute (Swiss TPH) and African Field Epidemiology Network (AFENET) and started training fellows in biosafety and biosecurity in 2013. GIBACHT provides theoretical knowledge on threats and existing prevention and copying strategies. It was funded by the German foreign office. In 2018, it recruited its fourth cohort and offered training to people who were based in universities, research institutions like laboratories, district local governments, ministries of health and non-governmental organizations from sub-Saharan Africa, North Africa, Middle East and Central Asia on biosafety and biosecurity.

To have a multiplier effect, the GIBACHT fellows train people working in their particular institutions. They use case studies provided by the consortium focusing on developing knowledge and skills in biosafety and biosecurity. Such training could contribute in closing those gaps among frontline health workers [8]. To prepare appropriately for subsequent trainings conducted at organizations where fellows come from, a training needs assessment questionnaire was designed to be filled by the participants. The aim was to explore the need for and possible barriers to biosafety and biosecurity training. This would inform the GIBACHT program on the training needs in these organizations and barriers to prepare their fellows for during the fellowship.

## Methods

**Study area:** the survey was conducted by the GIBACHT fellows during trainings conducted in their home organizations as part of the GIBACHT fellowship program. Responses were compiled from fellows from sub-Saharan Africa, Northern Africa, the Middle East and Central Asia. In 2018, workshops took place in five countries in sub-Saharan Africa, two in Northern Africa and three from Central Asia with each country having one workshop. In 2019, workshops were in four sub-Saharan African countries, four in North Africa, four in Central Asia and two in the Middle East.

**Study design:** this was a cross sectional assessment using qualitative and quantitative methods. A self-administered interview guide was used to explore reasons for and barriers to biosafety and biosecurity training. Findings were put in a self-administered questionnaire to quantify the findings and assess priorities identified by the respondents.

**Study population:** Table 1 indicates the number of respondents, the education status and the level of deployment of respondents during the trainings of GIBACHT fellows. There were 183 respondents in 2018 and 191 respondents in 2019. In 2019, questionnaires from 12 respondents were discarded because they had big gaps among the variables collected. Workshop participants were selected by fellows in collaboration with their supervisors from their own organizations. The training was done using a case study showing how a virus infection went global. Respondents for the training needs assessments were those who participated in these trainings. Majority of the respondents in both years had at least a bachelor’s degree. In 2019, more than half of the respondents worked at central level or higher compared to 2018 when they were slightly less than a quarter.

**Table 1:** respondents’ characteristics

Item	Variable	2018 N=183 (%)	2019 N=191 (%)
Sex	Female	82 (44.8)	76 (39.8)
	Male	101 (55.2)	115 (60.2)
Age of the respondent	19-25	55 (30.1)	23 (12.0)
	26-35	57 (31.2)	75 (39.3)
	36-45	40 (21.9)	74 (38.7)
	>45	31 (16.9)	19 (9.9)
Highest level of training	Certificate/diploma	22 (12.0)	11 (5.8)
	Bachelor	94 (51.4)	113 (59.2)
	Masters level	52 (28.4)	44 (23.0)
	Doctoral level	15 (8.2)	23 (12.0)
Level of deployment	Central or above	41 (22.4)	104 (54.5)
	District	56 (30.6)	18 (9.4)
	Regional	27 (14.8)	42 (22.0)
	Student	59 (32.2)	27 (14.1)

**Data collection:** data was collected by GIBACHT fellows while conducting trainings from April to July 2018 and April to June 2019. GIBACHT fellows were epidemiologists, biologists and medical professionals who have a first degree, but most had a masters’ degree or PhD and they were working in universities, ministries of health or research institutions. In these two years, there were 15 female and 18 male fellows. Workshop participants would first be given an explanation for the training needs assessment and those who consented would be given questionnaires. Only respondents and GIBACHT fellows would be present during the filling of the questionnaires. Tools were pre-tested among the GIBACHT fellows during a training workshop prior to collecting the training needs assessments and adjustments made. Questionnaires in both years were self-administered and were filled and completed during the training sessions which took between two to three hours. In 2018, a structured questionnaire collecting data on age and sex of the participants, completed level of education and deployment level was used. Respondents would indicate whether they support training in biosafety and biosecurity at their institutions and the barriers to such a training. Questionnaires were distributed either at the beginning or at the end of the training, but the collection was done at the end. Questionnaires were in English and for those countries which needed translation, they were first translated into the language commonly understood by the participants. Responses were given in the language of translation and answers translated back into English. There were no self-identification data on the questionnaire. In 2019, the barriers identified from the structured questionnaire in the previous year were quantified using a Likert scale to assess whether the participant strongly agreed, agreed, neither agreed nor disagreed, disagreed, or strongly disagreed with them. The tool was pre-tested among GIBACHT fellows during a training workshop and adjustments made.

**Data management and analysis:** hard copies of the filled questionnaires were collected by fellows and submitted to the program in July 2018. The qualitative responses were typed verbatim from the questionnaires into a word file. The qualitative responses were coded and analysed using manifest content analysis [9]. They were later grouped into themes and the linkage between the themes determined. For barriers against such training, responses were grouped according to building blocks of the health system framework namely governance, financing, human resources, information system, supplies and service delivery. Preliminary findings were presented to the GIBACHT fellows in July 2018 and modifications made accordingly. In 2019, answers of the respondents in various organizations were filled into an excel sheet and together with the hard copies submitted to the GIBACHT program. The excel sheets were anonymized by removing the organizations’ names and entered into Epi-info 7 (Centers for Disease Control and Prevention, Atlanta, Georgia) for analysis. Results are given in tables and charts. Preliminary findings were presented to the GIBACHT fellows in July 2019.

**Ethical considerations:** all respondents were given an explanation of the objectives of the training needs assessment and those who did not want to fill the questionnaires were free not to fill them. All the personal identification data was not required on the questionnaires and was not collected. Ethical clearance to use this programmatic data was given by Makerere University School of Public Health Higher Degrees and Ethics Committee.

## Results

Respondents supported training in biosafety and biosecurity because of individual, community and environment benefits. Barriers included lack of finances, governance issues, human resource constraints, inadequate information and lack of equipment and infrastructure.

**Figure 1:**
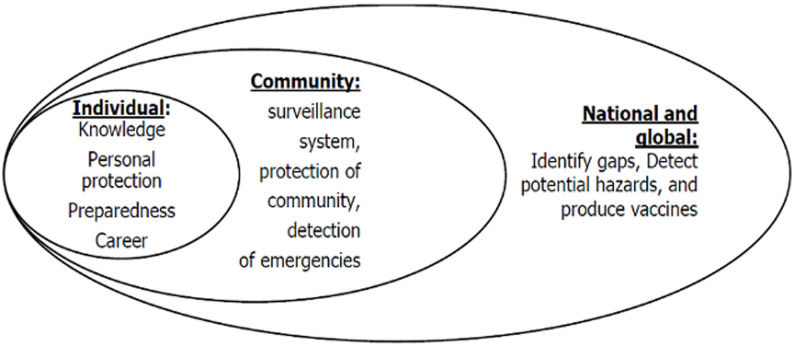
rationale for training in biosafety and biosecurity

**Supporting training in biosafety and biosecurity:** in 2018, a total of 176/183 (96.17%) supported training in biosafety and biosecurity. They cited individual, community and national or global benefits. These benefits were interlinked as depicted in Figure 1.

**Individual benefits:** many respondents especially students supported having training in biosafety and biosecurity because it would bring about capacity building. Such training would create awareness among trainees, impart skills in epidemic preparedness, improve handling of biological specimens, provide skills on how to use personal protective wear, make trainees aware of the dangers of research and create career interests in students or front line workers: “*Training in biosafety and biosecurity helps to equip one to effectively investigate an outbreak*” (master’s level - student); “*Biosafety and biosecurity training enhances surveillance on infectious diseases*” (bachelor’s level - student); “*There should be training in biosafety and biosecurity because it makes a trainee familiar with the basic steps and guidance one should follow during outbreaks*” (bachelor’s holder - central level); “*The training imparts knowledge on proper handling of organs or tissues of biological origin*” (certificate holder - regional level); “*It increases awareness of the spectrum of bio attacks from natural, the unintentional to intentional*” (bachelor’s level - student); “*There should be training in biosafety and biosecurity because it creates interests of medical students and health care professionals in the topic*” (doctorate holder - central level); “*If you want to save people’s lives, you have to protect yourself first. For this reason, you have to know all about biosafety and biosecurity and afterwards you can help people not to be infected*” (bachelor’s level - student).

**Community level benefits:** other reasons given for training in biosafety and biosecurity were ensuring prevention of infection within the community or the workplace like laboratories. Most of those who highlighted protection of the community came from people working at districts or central government. They cited strengthening the surveillance system, the diagnostic capacity and the ability to detect an emergency. Such training would reduce unintentional exposure to pathogens or toxins and improve waste management. It would also improve skills of health workers so that they can train those at lower levels. The training would make the community safer. Knowledge would also be transmitted to other people: “*Training will assist in the control of contagious diseases at the community level*” (certificate holder - district level); “*Training helps to inform trainees on the need for the proper use of laboratory and laboratory equipment*” (bachelor level - student); “*As we are always in contact with biohazards and collect samples that may contain hazardous materials, it will increase our capacity and our skills*” (masters holder - district level); “*Training will enable me to coach other health workers at lower levels*” (masters holder - district level).

**National and global level benefits:** respondents also highlighted strengthening the health system, protection of the environment, addressing global security issues and promoting the good use of microorganisms in the production of vaccines. Training in biosafety and biosecurity would not only prevent spread of infectious diseases but also contribute to production of vaccines and research on treatment: “*Training helps in identifying gaps to strengthening biosafety in the country*” (doctoral level holder - central level); “*Biosafety and biosecurity training gives an understanding of potential biohazards in the environment*” (bachelor’s holder - regional level); “*In a global scenario, it is important to protect people from highly contagious diseases*” (masters holder - district level); “*Training is necessary to avoid the accidental release of harmful substances into the environment*” (master’s level - student); “*Training enhances the use of safe microbes for the production of vaccines*” (doctoral holder - central level).

**Not-supporting training in biosafety and biosecurity:** out of 183 respondents in 2018, seven people (3.83%) indicated that there should not be training in biosafety and biosecurity. The seven people were coming from six countries. One was a certificate level holder, four were at bachelor level and two had master’s level training. All those with training at the doctoral level indicated that there should be training in biosafety and biosecurity. Those who said there should not be biosafety and biosecurity training were not opposed to it as such. They either did not see themselves benefiting from such training or thought it was already incorporated into the existing training. Some respondents thought that biosafety and biosecurity was not applicable at their level of employment or where they intended to work: “*There is no concept (of biosafety and biosecurity) at the district level. It should be a priority at the national level*” (masters holder - district level); “*I guess I won’t work with all these things. So that is why I have no reason to learn it*” (bachelor’s level - student); “*The training should be just for laboratory staff*” (bachelor’s level - student). Others thought it was already being incorporated into the existing training hence what was needed is not a new set of training but better implementation of what existed. “*Because it is already incorporated into fundamental laboratory methods as a stand-alone course at masters’ level. It only needs to be better structured and organized*” (master’s level - student).

**Barriers:** respondents indicated that there were financial, governance, human resource, information systems and infrastructure and equipment constraints. This cut across respondents from different employment levels and training background. Lack of policy or guidelines on biosafety and biosecurity was cited as a key barrier to training. In places where the regulations were present, they were poorly formulated and where they were well-formulated there was inadequate dissemination or implementation of guidelines leading to inadequate knowledge at the community level as well as lack of adherence to regulatory laws which enforce biosafety: “*There is less concern for biosafety and biosecurity at national level*” (masters holder - district level). Participants indicated that biosafety and biosecurity training was poorly financed and with budget constraints, there were inadequate resources for such training. This was the most common reason given across all the countries and GIBACHT fellows as exemplified in the following quote: “*The challenge is lack of financial resources amidst other competing priorities aside from biosafety*” (master’s level - student). Human resource constraints were cited by many of the respondents as a big hindrance to training in biosafety and biosecurity. This ranged from having inadequate numbers with limited numbers of highly qualified specialists. There was a lack of local experts and where they existed, they were overloaded because of being insufficient to handle the workload. Some indicated that there was no trainer of trainers (TOT) teams: “*The challenge is that there is lack of skills, personnel and expertise*” (masters’ level - student).

The information system was identified as another barrier. Some indicated that there were limited database and literature in some of the commonly used languages in those countries. There was lack of information about suspected organisms, lack of adequate information on biosafety, poor dissemination of various biosafety measures, or complete lack of dissemination of information. In some countries, an integrated public health system did not exist. In some places, it was an inadequate curriculum or lack of instructional materials and manuals: “*One of the barriers to the training in biosafety and biosecurity is lack of instructional materials, manuals…*” (doctoral holder - central level). Inadequate supplies and infrastructure was another barrier that was highlighted. Respondents cited lack of equipped laboratories, inadequate provision of personal protective equipment and low diagnostic capacity: “*There is lack of Personal Protective Equipment (PPEs) at the facility level and during outbreaks*” (masters holder - district level). In 2019, these qualitative perceptions were quantitatively assessed. There were 191 responses from fellows’ workshop participants that ranked on a Likert scale the barriers in governance, financing, human resources, information needs, infrastructure and supplies. Governance barriers were assessed using the maturity model spanning from no guidelines to lack of adherence to guidelines: having no policy guidelines on biosafety and biosecurity, poorly formulated guidelines, poorly disseminated guidelines or not adhering to guidelines. Financial barriers were assessed on whether the biosafety and biosecurity program was poorly financed or it was just the biosafety and biosecurity training that lacked financing.

**Figure 2:**
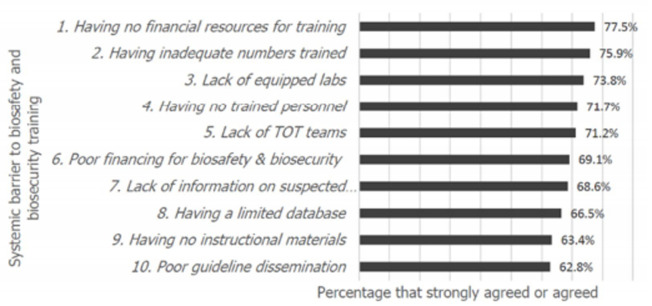
the top ten barriers with the highest agreement among the respondents

Human resource barriers were assessed at the presence or absence of trained personnel in biosafety and biosecurity but also the presence or absence of trainer-of-trainers (TOT) teams. Information and instructional materials were assessed on the absence or presence of a database of biosafety and biosecurity, relevant literature, information on suspected pathogens, curriculum and instructional materials. Infrastructure and supplies were assessed on whether there was inadequate infrastructure, lacking equipped laboratories or lacking provisions for personal protective wear. The results are indicated in Table 2. In governance, the largest gaps were having poorly formulated guidelines and poor guideline dissemination. Both the biosafety and biosecurity program broadly and the training in biosafety and biosecurity were perceived to be underfunded. Respondents also underscored the lack of trained personnel and lack of TOT teams in biosafety and biosecurity. Other major barriers included a limited database for biosafety and biosecurity, lack of information on suspected pathogens, having no instructional materials and inadequate infrastructure. In order to assess the highest-ranking barriers according to respondents, those who strongly agreed were added to those who agreed with the statements provided. The top ten barriers to training on biosafety and biosecurity are shown in Figure 2. Barriers cut across various systemic areas. Poor guideline dissemination came up. Poor financing for the biosafety and biosecurity program and poor financing for training in biosafety and biosecurity were key financial challenges. Lack of equipped laboratories, having no trained personnel and lack training of trainers (TOT) teams came up as major barriers. Information on suspected pathogens, having limited databases and lack of instructional materials were key informational challenges.

**Table 2:** perceptions of respondents on systemic barriers to biosafety and biosecurity training

Barrier to biosafety and biosecurity training	Strongly agree	Agree	Neither agree nor disagree	Disagree	Strongly disagree
**Governance**					
No policy guidelines	34	55	41	45	16
Poorly formulated guidelines	26	90	46	25	4
Poor guideline dissemination	25	95	56	13	2
Not adhering to laws	26	70	69	17	8
**Financial barriers**					
Biosafety/biosecurity program poorly financed	52	80	36	14	9
No finances for training	53	95	32	9	2
**Human resources**					
No trained personnel	47	90	21	29	4
Inadequately numbers trained	49	96	24	18	4
Heavy workload	25	77	74	13	2
Lack of TOT teams	51	85	28	15	12
**Information and instructional materials**					
Limited database	32	95	43	18	3
No biosafety and biosecurity literature	36	71	35	42	7
Lack of information on suspected pathogens	27	104	28	29	3
No curriculum on biosafety and biosecurity	46	67	53	19	6
No instructional materials	41	80	38	26	6
**Infrastructure and supplies**					
Inadequate infrastructure	35	81	48	21	6
Lack of equipped laboratories	34	107	30	17	3
Lack of provision of PPEs	25	85	24	47	10

## Discussion

Majority of respondents supported training in biosafety and biosecurity. Reasons given focused on increasing knowledge and skills of trainees, protecting the community or the immediate work environment and strategically protecting the environment and making good use of microorganisms to produce vaccines. Barriers were systemic. There were governance, financial, human resources, information system, equipment and infrastructure challenges. Many respondents especially students supported training in biosafety and biosecurity due to individual capacity building and career progress. Capacity building addresses challenges of changing patterns of zoonotic diseases [10] like the one health approach [2,11]. Training could be embedded in pre-service curricula of those intending to work in disease prevention and control. However, as disease patterns keep changing, those already in working positions need also training in biosafety and biosecurity within the framework continuous professional development. In a study done in Korea, it was realized that several healthcare workers (HCWs) were infected with MERS-CoV during the MERS-CoV outbreak, with the major contributing factor being inadequate training in biosafety [12]. Capacity development in biosafety and biosecurity of personnel already in the workforce has been demonstrated by the NUITM-KEMRI biosafety training program over the years, with an aim of equipping laboratory personnel prior to their commencement of work at a biosafety level 3 laboratory [13]. Protection of the community and the immediate workplace was the rationale for biosafety and biosecurity training especially by those working at operational levels like districts.

In 2014, more than 170 health care workers (HCWs) were infected in the west African ebola outbreak with several of them dying, signifying that, HCWs were at risk due to their working environment [14]. The WHO also stated that HCWs were 21-32 times more at risk of contracting ebola, compared to ordinary adults in Guinea, Sierra Leone and Liberia [15] due to their risk of exposure at the workplace. This necessitated training in biosafety and biosecurity of HCWs organized by the WHO and the Centres for Disease Control and Prevention [16]. It is critical to appreciate that in-service training contributes to protection of the immediate workplace. Training in biosafety and biosecurity was also justified for strategic reasons like national or global security and environmental protection. Increasing capacity among workers at the frontline helps to stop diseases spreading globally [17]. Similarly, biosafety protects the populace at every level from global to family level [18]. Though laboratory research, pharmaceutical and vaccine development are of great benefits to disease control, poor handling could pose a danger to laboratory staff, the community and the world at large [19]. Poor guideline dissemination and other governance issues were challenges to biosafety and biosecurity training. Institutional commitment is important for biosafety [20]. In a study among Ugandan cattle farmers on perceived strategies to improve biosecurity, participants mentioned that absence of support from government, non-governmental organizations (NGOs) and other partners affected their adequate practice of biosecurity [21]. Another study conducted among workshop attendees on biosafety and biosecurity in Pakistan also revealed nonexistence of national regulatory control [22].

It is critical that international and national biosafety and biosecurity conventions and guidelines be widely disseminated so that institutions that deal with biosafety and biosecurity are aware. Lack of finances for a biosafety and biosecurity program or training were highlighted by a big percentage of the respondents as a barrier. In a study conducted among stakeholder organizations working in the UK natural environment, finance was highlighted as a barrier to the uptake of biosecurity [23]. Financial challenges have also been identified as barriers to establishing a biosafety level 3 (BSL-3) lab in low-income countries [24]. In Pakistan, increased cost of biosafety regulation execution were identified as a barrier in biosafety and biosecurity implementation [22]. This calls for government and other stakeholder commitments in terms of financial support in order to achieve successful implementation of biosafety and biosecurity. Lack of trained personnel and lack of trainers of trainers in biosafety and biosecurity programs were also highlighted as major barriers. The need for biosafety and biosecurity training has been shown in various countries [13,25]. Skills imparted need to reach the frontline actors where the first contact with pathogens take place. Some trainings at this operational level have not been taken up by the farmers with enthusiasm [26,27]. Trainers of trainers’ programs would ensure continuation of training for longer periods of time sustaining consistent training. Lack of information on certain pathogens, a limited database and instructional materials were identified as barriers to the implementation of biosafety and biosecurity. Efforts to generate locally adopted training materials outside high income countries have been reported [28].

States are mandated to conduct biosafety and biosecurity training to prevent any unintentional spread of infectious diseases [29]. Support across different nations is critical to avert any global pandemic. It is important therefore that institutions share instructional materials to increase capacity across the globe as a way to build an infrastructure that would identify a pathogen at source and control its spread to other areas. Limited infrastructure and supplies was also mentioned by many respondents as a barrier to biosafety and biosecurity training. Governments and other organizations must be committed to providing the necessary equipment, logistics and other infrastructure in order to effectively execute biosafety and biosecurity practices. In a qualitative study conducted in Brazil to determine adherence to measures, participants reported that limited infrastructure was a contributing factor to their inability to adhere to good health practice [30]. In connection with the revised laboratory biosafety manual, “core requirements” have been proposed emphasizing laboratory infrastructure and other equipment [31]. Training programs like GIBACHT contribute to enhancing knowledge and skills in in-service professionals and bring out teams of trainers of trainers. They also address some of the identified barriers like provision of training materials and information on databases. Unfortunately, there are other barriers that cannot be addressed by a training program like offering finances for a biosafety and biosecurity program or equipping laboratories. Highlighting such information to appropriate partners may help generate needed resources. There is diversity of capabilities across different organizations but convergence on common barriers indicates that certain gaps cut across many organizations in different countries.

**Strengths and limitations of the study:** the strength of this data is that it cuts across many countries and organizations and highlights what could be possible gaps in training for biosafety and biosecurity. Secondly, by combining exploratory and quantitative methods of data collection, the manuscript highlights both the scope of possible challenges as well as quantifying priorities according to respondents. The limitation is that this is programmatic data, hence it is not representative of the countries in the regions. Each organization or country needs its own training needs assessment to articulate its specific needs.

## Conclusion

Support for having biosafety and biosecurity training is high. Different organizations have different capabilities to handle biosafety and biosecurity events. Organization’s gaps need to be individually assessed. Barriers differ across organizations. Gaps go beyond what training programs can provide. Government commitment is vital particularly in terms of finance and legislation to guide biosafety and biosecurity training implementation.

### What is known about this topic

- There is increasing potential for zoonotic epidemics stemming from increased human-animal interaction and travel;
- There is lack of awareness of biosafety and biosecurity among people working in laboratories who are handling agents of infectious diseases;
- There is inadequate training for people at the frontline in farms and other areas where possible global pandemics could start from.

### What this study adds

- There is strong support for biosafety and biosecurity training across people working at different levels and with different educational backgrounds in different countries;
- There are systemic barriers to biosafety and biosecurity training which need to be addressed in order to have effective training;
- Whereas there are common barriers that cut across different organizations, there are also differences and each organization needs a comprehensive needs assessment done to identify its unique gaps.
